# ProSim: A Method for Prioritizing Disease Genes Based on Protein Proximity and Disease Similarity

**DOI:** 10.1155/2015/213750

**Published:** 2015-08-03

**Authors:** Gamage Upeksha Ganegoda, Yu Sheng, Jianxin Wang

**Affiliations:** School of Information Science and Engineering, Central South University, Changsha 410083, China

## Abstract

Predicting disease genes for a particular genetic disease is very challenging in bioinformatics. Based on current research studies, this challenge can be tackled via network-based approaches. Furthermore, it has been highlighted that it is necessary to consider disease similarity along with the protein's proximity to disease genes in a protein-protein interaction (PPI) network in order to improve the accuracy of disease gene prioritization. In this study we propose a new algorithm called proximity disease similarity algorithm (ProSim), which takes both of the aforementioned properties into consideration, to prioritize disease genes. To illustrate the proposed algorithm, we have conducted six case studies, namely, prostate cancer, Alzheimer's disease, diabetes mellitus type 2, breast cancer, colorectal cancer, and lung cancer. We employed leave-one-out cross validation, mean enrichment, tenfold cross validation, and ROC curves to evaluate our proposed method and other existing methods. The results show that our proposed method outperforms existing methods such as PRINCE, RWR, and DADA.

## 1. Introduction

Disease gene prioritization aims to suggest potential implications of genes in disease susceptibility. Also, it is important to know genes that are related to a particular disease in order to treat it. Hence identifying the genes related to a specific disease is one of the major challenges in the field of bioinformatics. To do so it is vital to consider biological details such as biological functions, patterns of expression in different conditions, and interactions with other genes. Furthermore, it is important to know functional annotations of candidate genes to a disease or phenotype under investigation as there are close relationships between the biological aspects and related diseases.

In medical research it is necessary to understand the genetic background of diseases with major implications in order to diagnose, treat, and develop drug for these diseases. Linkage analysis and association studies are some of the traditional gene-mapping approaches that have demonstrated remarkable success in this field [1]. Family-based linkage analysis is able to correlate diseases with specific genomic regions. Experimental examination of causative mutations in genomic regions is expensive and laborious as it consists of hundreds of genes. Thus to handle this challenge, traditional approaches are more time-consuming and costly while computational approaches are often considered to offer more efficient and effective alternatives. Therefore computational approaches have been developed to prioritize candidate genes for a particular disease. Different approaches have used various data sources such as gene expression [2, 3], sequence similarity of genes, DNA methylation [4], tissue-specific information [5], functional similarity and annotations [2, 6], and protein-protein interactions (PPIs) [7, 8] in determining the strength of association between genes and diseases as well as associations between diseases and protein complexes [9]. Network-based prioritization methods [10] are based on the observation that genes related to similar diseases tend to lie close to one another in the PPI network [11].

Furthermore, some other researchers have considered phenotype similarity in terms of gene closeness to prioritize disease genes. Depending on these studies, the correlation between phenotype similarity and gene closeness, defined by a concordance score, is a strong and robust predictor of disease genes [12]. Meanwhile, some researchers used tissue-specific gene expression data along with PPI networks to prioritize disease genes as many disorders are involved a disruption of the “molecular fabric” of different, healthy tissues [12]. In addition, some used support vector machine recursive feature elimination (SVM-RFE) method for gene selection in different cancer tissues by incorporating a minimum-redundancy maximum-relevancy (MRMR) filter [13]. In current studies, phenotypic similarity between the diseases of interest to other diseases for which causal genes are known has been used to prioritize candidate genes [3, 14]. Simultaneously, some researchers grouped diseases into separate disease families to facilitate the prioritization task [15]. Topological properties of PPI networks are also used to understand genetic diseases [3, 8, 16] and essential proteins [17, 18]. Gonçalves et al. combined full topology scores which were computed by using local clustering on graphs or diffusion kernels over confidence weighted gene association networks by integrating evidence from heterogeneous sources, in order to prioritize disease genes [19]. Furthermore, special local clustering has been used to identify genes associated with Alzheimer's disease. With the use of special local clustering algorithm it is able to group genes together with similar expression patterns and identify significantly varied gene expression values as isolated points [20].

This study proposes a new algorithm called proximity disease similarity algorithm (ProSim) which combines protein proximity in PPI networks and disease similarity into a single mathematical formula used to prioritize candidate genes. According to the previous work, protein proximity shows that genes close to the true disease genes tend to be disease genes as well in PPI networks. Meanwhile, disease similarity provides details of how query disease is related to other diseases with regard to phenotypic characteristics. This is expected to increase the power of prioritizing candidate genes to a relevant query disease. The proposed algorithm is evaluated on six case studies and its performance is compared with other existing methods. The results show that the proposed method is superior to existing methods.

## 2. Materials and Method

### 2.1. Materials

PPI networks: positive PPI network is downloaded from the Human Protein Reference Database (HPRD). It consists of 9673 nodes with 39240 edges. Negative PPI network is downloaded from the Negatome database, which consists of 1828 nodes with 2171 edges.

Tissue-specific gene expression data are downloaded from Gene Expression Omnibus (GEO) in the National Center for Biotechnology Information (NCBI) website (GEO accession number GSE 7307).

### 2.2. Methods

**Figure 1:**
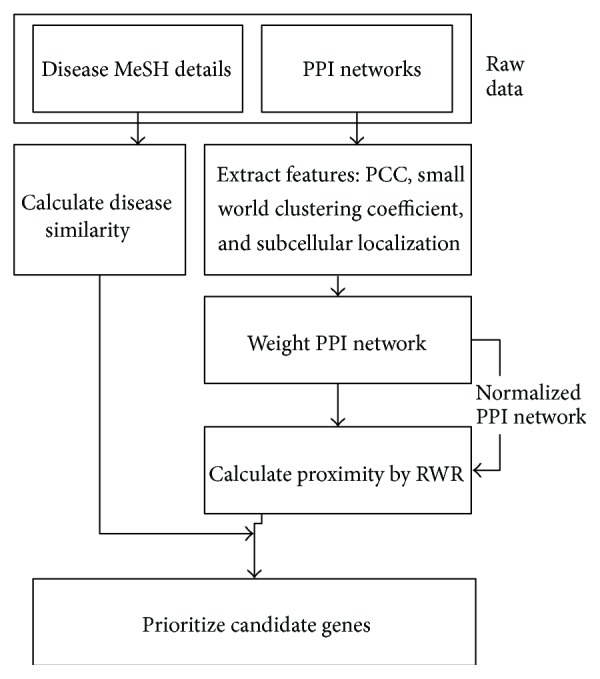
Flow chart of ProSim method.

The proposed method consists of five main steps described in detail in the following five subsections. The first subsection describes the feature extraction process in which three main features are used to evaluate the effectiveness of the PPIs in positive and negative PPI databases. Next a logistic regression function in these three features is described. The third subsection explains how random walk with restart method is used to calculate the topological similarity of the proteins in the PPI network. The fourth subsection describes how disease similarity is calculated. The fifth subsection explains how all these details are then used to prioritize candidate proteins [21]. The entire process is illustrated as a flow chart in Figure 1.

#### 2.2.1. Feature Extraction

As mentioned in Materials, two kinds of PPI networks are employed in this study: positive and negative PPI networks from which three types of features have been extracted as described in the sequel.

*(1) Small World Clustering Coefficient.* Small world networks have high clustering coefficients. The cliquishness of the neighborhood around an individual edge should therefore be an indication of how well this edge fits the pattern of a small world network. In order to calculate the clustering coefficient of proteins *v* and *u*, the following equation was used:

(1) $$\begin{array}{c} C_{vu}=-\log\overset{\min\{\left.\left|N(v)\right|,\left|N(u)\right|\right\}}{\underset{i=\left|N(v)\cap N(u)\right|}{\sum }}\frac{\left(\begin{array}{c} \left|N\left(v\right)\right|\\ i \end{array}\right)\left(\begin{array}{c} N-\left|N\left(v\right)\right|\\ \left|N\left(u\right)-i\right| \end{array}\right)}{\left(\begin{array}{c} N\\ \left|N\left(u\right)\right| \end{array}\right)}, \end{array}$$

where *C*_*vu*_ denotes the small world clustering coefficient, *N*(*v*) and *N*(*u*) denote the sets of proteins that directly interact with proteins *v* and *u*, respectively, and *N* is the total number of proteins in the network. By using the above formula, a small world clustering coefficient is calculated for each pair of proteins in both positive and negative PPI networks.

*(2) Pearson Correlation Coefficient.* Pearson correlation coefficient (PCC) is used to calculate coexpression measurements for corresponding genes derived from multiple sets of tissue-specific microarray experiments [22]. PCC values are used since coregulated genes are more likely to interact with each other compared with other genes [23, 24]. Gene PCCs are mapped to corresponding proteins. In this study, the correlation coefficient quantifies the similarity of expression between two genes and shows whether the corresponding proteins interact or not [11, 25]. Let *v* and *u* be two m-dimensional vectors representing expression profiles of two genes which correspond to proteins *v* and *u*, respectively. $\overset{-}{v}$ and $\overset{-}{u}$ are the mean of *v* and *u*, while *σ*_*v*_ and *σ*_*u*_ are the standard deviations of *v* and *u*, respectively. PCC is calculated for both positive and negative PPI networks by using

(2) $$\begin{array}{c} \rho_{vu}=\frac{\left(1/m\right)\sum_{i=1}^{m}v_{i}u_{i}-\bar{v}\bar{u}}{\sigma_{v}\sigma_{u}}. \end{array}$$

*(3) Protein Subcellular Localization.* The last feature is a protein subcellular localization data of interacting partners. Protein subcellular localization data of interacting partners is represented by either zero or one [26]. To identify the subcellular location of each protein Hum-PLoc technique was used [27]. Hum-PLoc is a predictor developed by Shen and Chou that is able to deal with the multiplex problems of the human protein system. As such, the coverage scope for human proteins is extended from 4 to 12 location sites. The subcellular location sites are cytoplasm, mitochondria, nucleus, plasma membrane, centriole, cytoskeleton, endoplasmic reticulum, extracell, Golgi apparatus, lysosome, microsome, and peroxisome. In order to get the subcellular location, all the protein sequences should be obtained first. To get the sequences of proteins in positive and negative PPI networks proteins are mapped from the Swiss-Prot database. Thereafter, protein sequences are used with Hum-PLoc predictor to identify the subcellular location of that particular protein. Huh et al. [28] note that not every subcellular location has a biological significance with others. They publish a list showing which subcellular locations have a biological significance with each other. Based on the list, every pair of proteins in PPI networks is assigned a value of 1 if listed and 0 otherwise. The value is purely based on the fact of biological significance of each subcellular location without considering whether protein interaction actually exists or not.

All these three features are then used in a logistic regression function to calculate a reliability score for each protein in the network [26].

#### 2.2.2. Logistic Regression Function

A logistic regression function is employed to calculate the weight of each interaction in the PPI network. To train the logistic regression model we have used HPRD as the golden standard positive PPI network and Negatome database as the negative PPI network. According to the logistic distribution, the probability of true interaction *T*_*vu*_ given the three input features *X* = (*X*_1_, *X*_2_, *X*_3_) is calculated based on the formula as follows:

(3) $$\begin{array}{c} \mathrm{Pr}\left(T_{vu}\;|\;X\right)=\frac{1}{1+\exp(-\beta_{0}-\sum_{i=1}^{3}\beta_{i}X_{i})}. \end{array}$$

According to the general logistic regression function it is better if the positive and negative datasets are balanced in order to obtain the best values for regression constants. For this purpose we have considered only a portion of the positive PPI network whose size will be equal to that of the negative PPI network. For the simplicity we have selected first 2000 interactions from the HPRD and almost the same amount of interactions from the negative PPI network as well.

#### 2.2.3. Random Walk with Restart to Calculate the Proximity

For a given disease *q*, in order to calculate the proximity between proteins, two sets of genes, seed set *S* and candidate set *C*, are used. A seed set *S* consists of genes known to be associated with the query disease *q*. A candidate set *C* specifies one or more genes, “potentially” associated with the disease *q*, created by excluding the seed genes from the PPI network. The rest of genes related to the given PPI are then considered as the candidate set.

Random walk with restart method is used to compute the proximity of candidate genes to relevant seed genes. Random walk with restart method is actually a generalization of Google's well-known page-rank algorithm [29]. After getting the proximity values for each protein in the network, the values are then sorted in a descending order. These proximity values are used at the final stage of the process, to prioritize the candidate proteins.

#### 2.2.4. Calculation of Disease Similarity

Disease similarity is calculated based on a metric proposed by van Driel et al. [14], who used the medical subject headings vocabulary (MeSH) to extract terms from Online Mendelian Inheritance in Man (OMIM) to identify similar diseases. Each MeSH entry is a collection of terms with synonyms and plurals, called a concept. MeSH provides a standardized way to retrieve information that uses different terminologies to refer to the same concepts. van Driel et al. tested the prediction power of different ranges of similarity values by calculating the correlation between the similarity of two diseases and the functional relatedness of their causal genes. According to their research findings, similarity values in the range [0, 0.3] were not informative while genes with similarities in the range [0.6, 1] showed significant functional similarity. A logistic function *L* shown in (4) is used to calculate a probability that two diseases are related:

(4) $$\begin{array}{c} L\left(x\right)=\frac{1}{1+e^{\left(cx+d\right)}}. \end{array}$$

The values for the parameters *c* and *d* were set as *c* = −15 and *d* = log⁡(9999). *L* was then used to compute the prior knowledge to a particular disease, denoted by *Y*, as shown in

(5) $$\begin{array}{c} Y\left(q\right)=L\left(S\left(p,q\right)\right), \end{array}$$

where *q* is the query disease and *S*(*q*, *p*) is the similarity between diseases *q* and *p*. This equation is slightly different from the way of prioritization and complex elucidation algorithm (PRINCE) [30] to calculate the disease similarity. Here disease similarity is calculated as a global representation. The original PRINCE algorithm showed how each protein in the PPI network was associated with different diseases. Thus if disease *p* was more similar to disease *q*, disease *p* was selected.

#### 2.2.5. Final Stage of the Process

At the final stage a prioritization score was calculated. In the proposed approach PRINCE algorithm was modified by incorporating both proximity and the disease similarity values as shown in

(6) Fv=α∑u∈N(v)Fuw′(v,u)+1−αYq+Pro(v),

where *F*(*v*) reflects the relevance of protein *v* to disease *q*. The prioritization function consists of three inputs: given a PPI network *G* = (*V*, *E*, *w*), where *V* represents the set of proteins, *E* is the set of interactions, and *w* denotes the weight of each interaction, a normalized form of adjacency matrix *w*, denoted by *w*′, is derived and used as one of the inputs. The adjacency matrix is constructed by using the reliability score obtained from the logistic regression function discussed in Section 2.2.2. The matrix has been normalized with the weight of an edge by the degree of its end-points. The second input gives the prior information of disease similarity in relation to the query disease *q*. The calculation of disease similarity is explained in Section 2.2.4. The third input value is the proximity of proteins related to the seed genes. Proximity is calculated by using the random walk with restart method. In the proposed equation, *w*′ is a |*V*| × |*V*| matrix, while *F*, *Y*, and Pro are displayed as vectors of size |*V*|. To improve the execution speed and ensure a convergence of (6), a propagation based approach similar to work reported by Zhou et al. [31] is applied. The resultant equation (7) is thus guaranteed to converge after* enough *iteration:

(7) $$\begin{array}{c} F^{t}=\alpha W^{\prime }F^{t-1}+\left(1-\alpha \right)\left(Y\left(q\right)+\text{Pro}\left(v\right)\right). \end{array}$$

From (7), it can be seen that if a node has prior information, it will propagate the information to its neighbors. The process continues until the value converges or the maximum iteration value, *T*, is reached. In this study *T* is set to 100 and values of *α* varied in (0, 1).

## 3. Results and Discussion

This section details experiment results for the proposed method and also provides comparisons with existing methods. In particular, the proposed method has been evaluated via six diseases: breast cancer (MIM: 114480), colorectal cancer (MIM: 114500), lung cancer (MIM: 211980), prostate cancer (MIM: 176807), Alzheimer's disease (MIM: 104300), and diabetes mellitus type 2 (MIM: 125853). Performance comparison was done against the original PRINCE algorithm [30], random walk with restart (RWR) [24], and degree-aware disease algorithm (DADA) [29].

As indicated in Section 2.2.1, the first part of the experiment was to extract features of concern. Based on (1), the small world clustering coefficient of each protein interaction was calculated. Experiment results showed that most of the coefficients lay between −0.6 and −0.7. These high clustering coefficients of small world networks indicated that neighbors of a given vertex are more likely to have edges between them than expected. Next, PCC was calculated for each PPI. From the results PCC value was greater than 0.5 for most of the potential candidate genes selected for a specific disease while negative values for the same indicated that those proteins were less significant to a given query disease. Lastly, a protein subcellular localization data of interacting partners was determined, in order to find the biological significance of the subcellular localization. Results indicated that a large amount of proteins was biologically significant in a given PPI network.

These three features were then used as inputs of a logistic regression function (3). Based on a tenfold cross validation method, ten sets of values of *β* were obtained by the logistic regression function, shown as fold *i*, *i* = 1,2,…, 10, in Table 1. Under each fold, 4 values corresponding to *β*_0_ to *β*_4_ are shown. The average value of *β* was then taken as the value used to calculate the probability of true interaction *T*_*vu*_ given the three input features *X* = (*X*_1_, *X*_2_, *X*_3_), which are 1760.946913, 36.81855608, 6.593051189, and −2014.167331 for *β*_0_ to *β*_4_, respectively.

**Figure 2:**
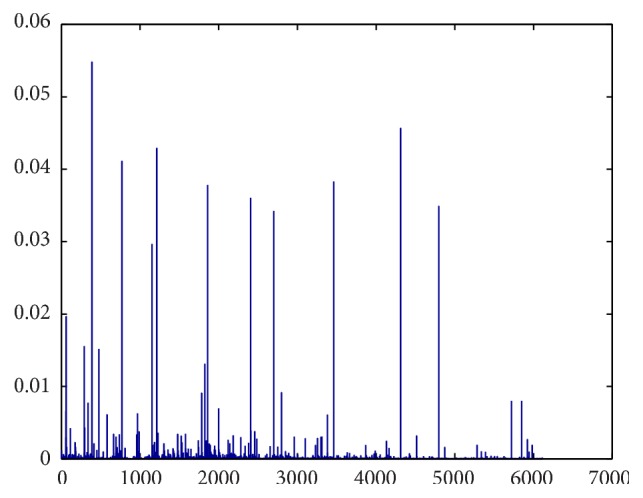
Proximity values for different proteins to prostate cancer.

Proximity values show proteins that are closer to a specific disease. Therefore if a proximity value is high, it indicates that the given protein has a high chance of being related to a given disease. Random walk with restartwas used to give proximity values. For example, Figure 2 shows proximity values of different proteins for prostate cancer disease. It illustrates around 25% of proteins in the network have high proximity value. As a result these proteins have a high prominent factor to be a disease related gene for a particular disease.

Disease similarity measures how similar a query disease was to other diseases. If the disease similarity value was less than 0.3 it meant that the diseases in question were not significantly relevant. When the value was higher than 0.6, a high connection existed between the given diseases.

**Figure 3:**
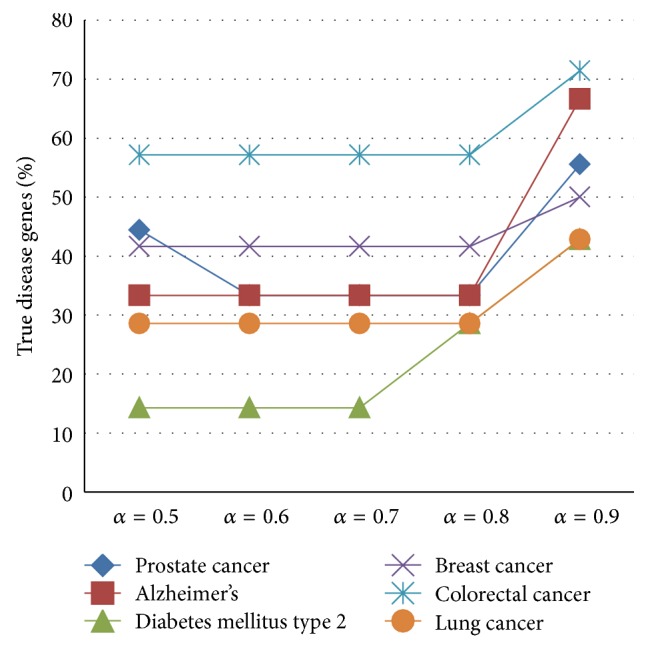
Alpha value prediction.

The final part of the experiment combined the proximity values with disease similarity values to calculate a prioritization score. Testing was carried out based on the prediction capacity of disease genes of the final equation in order to find out the best combination of proximity values with disease similarity. According to the results, by including the proximity value as an explicit value, the prediction capability will be increased. Furthermore, to tune the value of *α* used in (6), several values were experimented with for all the six diseases and the results are depicted in Figure 3. From Figure 3 the best results were obtained when *α* = 0.9.

**Figure 4:**
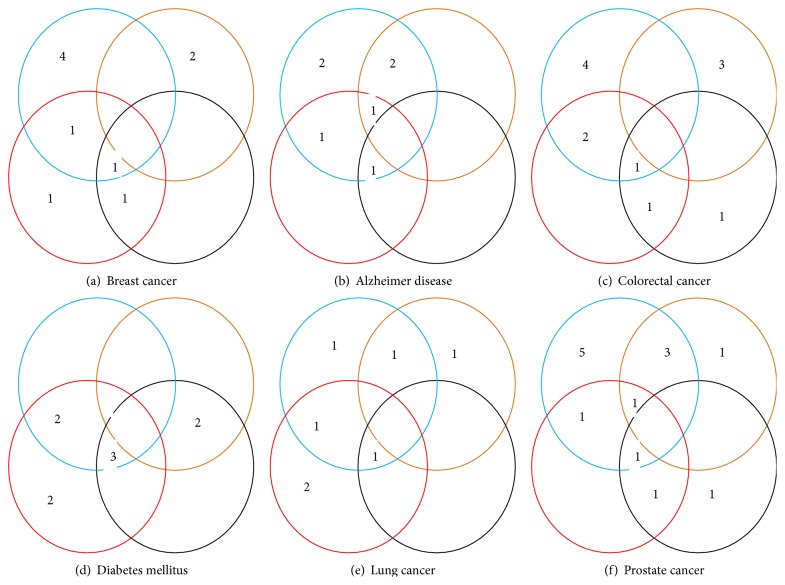
Comparison of top ten genes as a Venn diagram. Blue, orange, red, and black circles represent ProSim, PRINCE, RWR, and DADA methods, respectively. (a) Breast cancer. (b) Alzheimer disease. (c) Colorectal cancer. (d) Diabetes mellitus. (e) Lung cancer. (f) Prostate cancer.

The proposed method is compared with three other methods, namely, PRINCE, RWR, and DADA. By comparing the top ten genes ranked by each method one can conclude that the proposed method is able to predict more known and unknown disease genes than existing methods.  Table 2 shows the top ten genes predicted for each method and for each case study.  Figure 4 shows another representation of how much each method is able to predict known and unknown disease genes within the top ten disease genes.

To provide more performance comparisons of the proposed method to existing methods, a leave-one-out cross validation procedure was used. With each cross validation trial, a single seed gene related to the query disease was removed and then each method is evaluated on its success of identifying and ranking the removed seed gene. To replicate the case of prioritizing the proteins encoded by genes inside a linkage interval, an interval of size 100 was used similarly to the work by Köhler et al. [15]. But in this research, it will calculate the percentage of true disease genes identified within the top 100 genes. Hence the threshold value used to rank the candidate genes was set to 100.

As shown in Table 3 the proposed algorithm performed better than the other methods. The proposed algorithm identified true disease genes at 80%, 71%, 69%, 66%, 57%, and 50% for breast cancer, prostate cancer, Alzheimer's disease, colorectal cancer, diabetes mellitus, and lung cancer, respectively, significantly higher than all the rest; however, it was comparable to other methods on diabetes. Because the gene expression details used for the calculation of PCC are not affect to cause diabetes disease. Therefore it has given a negative impact on the final result. Hence this has given a direction to improve in the future work.

**Figure 5:**
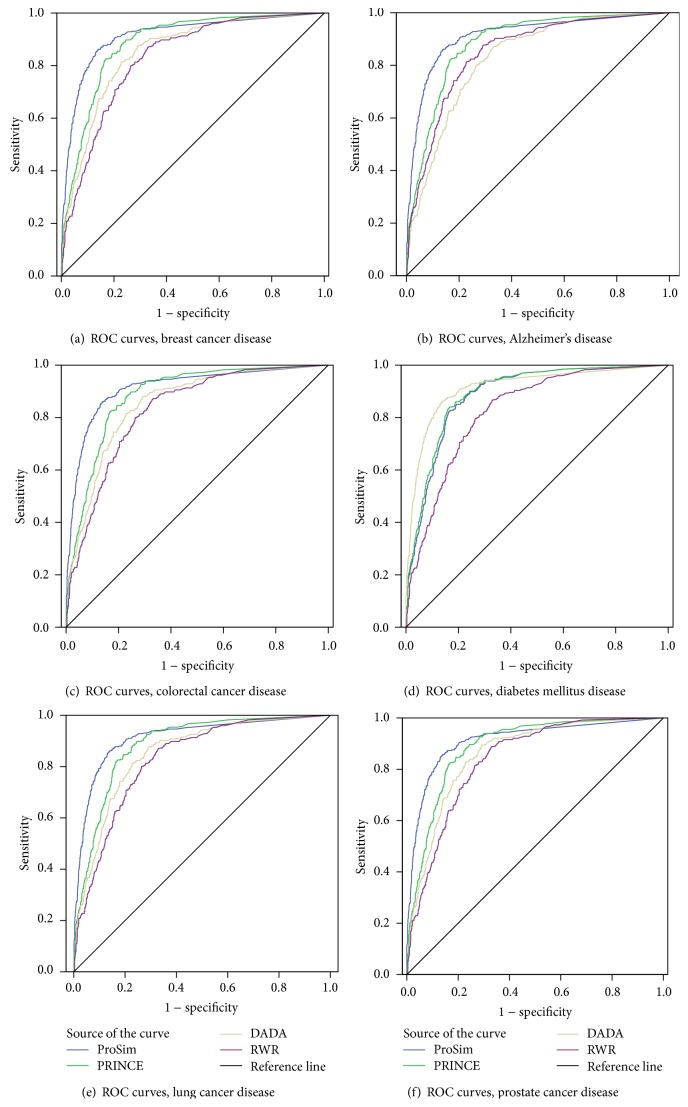
ROC curves: (a) breast cancer, (b) Alzheimer's disease, (c) colorectal cancer, (d) diabetes disease, (e) lung cancer, and (f) prostate cancer.

Further performance evaluation of the algorithm was based on sensitivity and specificity measures and used to draw ROC curves shown in Figure 5 for breast cancer, Alzheimer's disease, colorectal cancer, diabetes mellitus type 2, lung cancer, and prostate cancer, respectively. Sensitivity is defined as the percentage of true disease genes that are ranked above a specified threshold while specificity is defined as percentage of all nonrelated disease genes that are ranked below a specified threshold.

Lastly, a mean enrichment value [15] was calculated for each method and used for performance comparison purposes. In general, the mean enrichment formula is enrichment = 50/(rank). Based on ranking values, by using the leave-one-out cross validation process, it was possible to identify the rank of true disease genes for each method. The results are shown in Table 4. From the results, the proposed algorithm performed better than the other algorithms.

**Figure 6:**
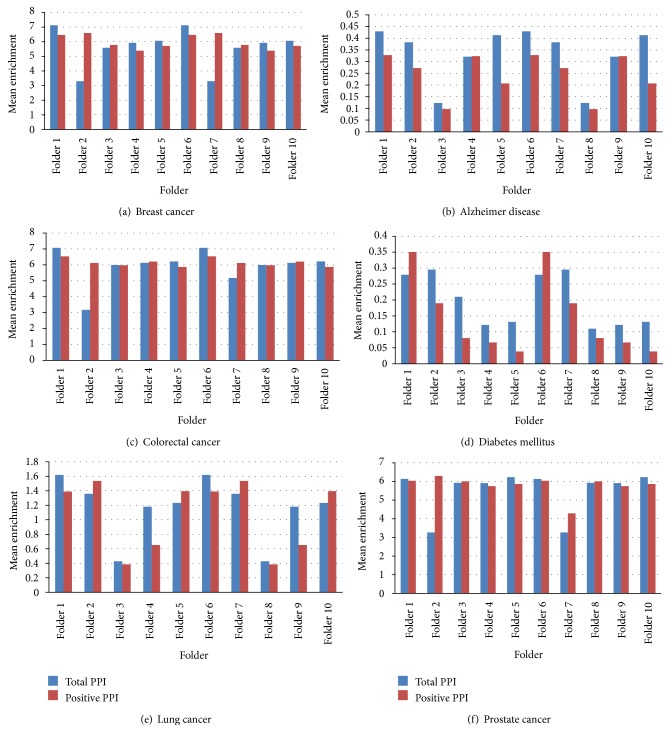
Tenfold cross validation for true disease gene prediction: (a) breast cancer, (b) Alzheimer disease, (c) colorectal cancer, (d) diabetes mellitus, (e) lung cancer, and (f) prostate cancer.

Tenfold cross validation is carried out to illustrate the performance of the proposed algorithm with the combination of positive and negative PPI networks as well as with positive PPI network. At each cross validation trial onefold PPIs were removed from the total PPI network and the rest of PPIs were used for the prioritization process. By calculation, onefold is one-tenth of total PPIs in the total network.  Figure 6 shows the results of the testing. These results show that the proposed algorithm is effective in identifying the correct disease related genes from all the positive and negative PPIs. Comparatively, the proposed method is able to predict disease genes with the positive PPI network, as well as the total positive and negative PPI network in an effective way.

The original PRINCE algorithm does not include subcellular localization data as a feature in calculating a reliability of the PPIs which could have impacted negatively on PRINCE in prioritizing of the candidate genes from the results of ProSim. Unlike the original PRINCE algorithm which included disease similarity unique to a specific PPI network, ProSim gives a global representation on how diseases relate to each other. In ProSim disease similarity is included as to how query disease relates to other disease. Hence, it will give more focus on the query disease than the original PRINCE algorithm, in which disease similarity is included for each protein in the PPI network. On the other hand random walk with restart method does not include disease similarity, which could have had a negative influence on their final result on detecting effective candidate genes for a specific disease.

Besides using different techniques to evaluate the performance of ProSim, it was important to identify the relevance of high ranked candidate genes to a given query disease. Thus, further evidence was sought from other online databases and scientific publications. By cross-checking predicted genes with other online databases and scientific publications it was found that HTT, SLPI, JUN, REL, and CD44 genes are tumor suppressors involved in several types of cancer, which were not predicted by the original PRINCE algorithm [32–36] yet identified by ProSim. ProSim also identified JUNB, MDM2 genes, which are used for therapy in prostate cancer [37, 38]. For Alzheimer's disease PSEN1 and JNK genes [39, 40] were ranked higher by the proposed algorithm. Finally, for diabetes mellitus type 2 disease, the proposed method ranked PIK3R1 and JUN genes [41] high. One important finding was that TP53 gene [35, 42] was ranked high except breast cancer and lung cancer as a common disease gene related to other diseases. When it comes to breast cancer, PPP1R13L, FOSL2, and ERBB4 were detected as new disease genes [43–45]. Furthermore, JUN, PIK3R1, and HTT genes were detected as tumor progression genes for colorectal cancer [46–48] and PPP1R13L gene for lung cancer disease [49]. In addition, for lung cancer disease it is able to detect some genes which affect therapy, such as ERBB4 and PLK1 genes [50, 51].

By considering the overall process, subcellular localization data, protein's small world clustering coefficient, and PCC of gene expression values gave a very good combination for calculating reliability of the PPIs. Furthermore experiment results showed that combining protein's proximity and disease similarity concepts resulted in improved performance in identifying and ranking candidate genes for a specific disease.

## 4. Conclusion

Prioritizing disease related genes is one of the important challenges in the field of bioinformatics. In order to address this challenge different computational methods have been introduced in past years. From the literature review it has been suggested that it is important to incorporate topological similarity with disease similarity in an algorithm for prioritizing genes related to a particular disease. This paper has proposed a new algorithm called ProSim. In this study, topological similarity is calculated using a random walk with restart method while disease similarity is calculated using the method introduced by van Driel. The performance of the proposed method is evaluated by comparing with three other methods, PRINCE, RWR, and DADA. Leave-one-out cross validation, mean enrichment, and ROC curves are the main evaluation techniques. Furthermore, the proposed method is able to predict disease genes effectively from a PPI network which consists of positive and negative PPIs. Last but not least it is able to identify some important candidate genes, previously ranked low by other methods, which include TP53, BRCA1, JUN, and PSEN1. Even though it outperforms existing methods considered, further experiments should be carried out to fine-tune its performance by including other biological data such as tissue-specific details as well as incorporating other mathematical procedures.

## Figures and Tables

**Table 1:** Values of *β* for each folder.

Fold 1	Fold 2	Fold 3	Fold 4	Fold 5	Fold 6	Fold 7	Fold 8	Fold 9	Fold 10
1058.461	1059.242	1058.926	1033.53	8125.654	1058.279	1058.381	1058.601	1069.272	1029.123
−0.04366	−0.03784	0.00557	1.690454	367.802	−0.02873	−0.02004	0.005051	−0.47721	−0.71004
0.875513	0.878244	0.877808	0.748819	58.37504	0.876113	0.877078	0.878938	0.873331	0.669624
−1166.52	−1167.42	−1167.09	−1140.27	−9689.94	−1166.34	−1166.48	−1166.75	−1178.31	−1132.55

**Table 2:** Top ten genes predicted for ProSim, PRINCE, RWR, and DADA.

Breast cancer				Colorectal cancer			
ProSim	PRINCE	RWR	DADA	ProSim	PRINCE	RWR	DADA
ATM	NBN	PIK3R1	PIK3R1	TP53	BRCA1	TP53	TP53
PPP1R13L	TIE1	HTT	FNTA	JUN	MSH2	AKT1	AURKA
FOSL2	BRCA1	CD44	ATM	PIK3R1	MLH1	AKT	RHOA
ERBB4	MSH2	CALM1	HTT	HTT	EP300	IL3	RET
BRIP1	PATZ1	JUN	SHOC2	NRAS	AKT1	NRAS	NTRK1
HIP1	DGCR2	MAPK3	RASSF2	PSEN1	BUB1B	BRCA2	CCND1
UBE2K	ATM	TPX2	CD44	CTNNB1	OGG1	BUB1B	BUB1B
RALGDS	MRE11A	SVIL	CALM1	CBL	RNF139	LYN	BRCA2
FOXG1	H2AFX	RAF1	JUN	AKT1	APC	AURKA	EP300
PLK1	TERF2	PSEN1	RAF1	VAV1	EXO1	HNF1A	GABRB1

Lung cancer				Diabetes mellitus			
ProSim	PRINCE	RWR	DADA	ProSim	PRINCE	RWR	DADA

PPP1R13L	TP53	PPP1R13L	IMPDH2	TP53	RHOA	AKT1	AKT1
ERBB4	VRK1	MAP3K8	APLP2	JUN	MAFA	PLN	INS
PLK1	TP53RK	IGF1R	PLK1	PIK3R1	BSCL2	PSEN1	PSEN1
BRD7	ERBB4	BRD7	ICMT	HTT	SLC2A2	LYN	JUN
UBE2K	CDKN2A	UBE2K	GSTM4	HNF1A	HNF4A	HNF1A	HNF1A
EGFR	TP53INP1	HUWE1	RAD17	PSEN1	INS	PCBD1	TP53
TAPBP	TAG	TAPBP	KLF4	LYN	IAPP	CASP8	ALB
UHMK1	EGFR	UHMK1	CASP8	CBL	NEUROD1	PIK3R1	NEUROD1
MAP3K8	RRM2	DGCR2	PIAS1	AKT1	GATA5	HTT	CEBPA
HIP1	CUL9	HIP1	FOSL2	VAV1	MAP3K13	EP300	RAC3

Prostate cancer				Alzheimer's disease			
ProSim	PRINCE	RWR	DADA	ProSim	PRINCE	RWR	DADA

TP53	NBN	TP53	TP53	TP53	HSD17B10	AKT1	AKT1
JUN	BRIP1	RNASEL	RNASEL	JUN	BACE2	PAX2	SFRP2
HTT	JUN	PAX2	AR	HTT	TP53	CASP8	PAX2
CD44	BRCA1	ERBB2	ABCE1	APBB2	JUN	PSEN1	CASP8
BARD1	HTT	CD19	AKT1	PSEN1	HADHB	APBB3	WT1
CD82	RAD50	BACE2	ERBB2	LYN	NAE1	BLMH	PSEN1
ERBB2	TP53	CASP8	CASP8	VAV1	APLP2	MAPK8	RAC3
REL	MRE11A	TSG101	BUB1B	CCND1	BLMH	WT1	RB1
SLPI	ATM	ATM	CCNE1	CASP8	APBB1	SFRP2	MAPK8
JUNB	BRCA2	CCND1	STAT5A	KIT	F12	RAC3	RHOA

**Table 3:** Fraction of true disease genes.

	ProSim	PRINCE	RWR	DADA
Breast cancer	**80%**	77%	72%	75%
Alzheimer's	**69%**	58%	55%	53%
Colorectal cancer	**66%**	63%	61%	62%
Diabetes mellitus type 2	57%	58%	53%	**69%**
Lung cancer	**50%**	48%	42%	45%
Prostate cancer	**71%**	61%	53%	60%

**Table 4:** Mean enrichment.

	ProSim	PRINCE	DADA	RWR
Breast cancer	**0.5565**	0.4896	0.393	0.2852
Alzheimer's	**0.1634**	0.1552	0.087	0.151
Colorectal cancer	**6.3233**	0.5926	3.6511	2.8521
Diabetes mellitus type 2	0.1998	0.246	**0.2414**	0.1488
Lung cancer	**0.2544**	0.1757	0.1625	0.1531
Prostate cancer	**6.2578**	3.0871	5.8649	2.386
